# Using complexity metrics with R-R intervals and BPM heart rate measures

**DOI:** 10.3389/fphys.2013.00211

**Published:** 2013-08-13

**Authors:** Sebastian Wallot, Riccardo Fusaroli, Kristian Tylén, Else-Marie Jegindø

**Affiliations:** ^1^Interacting Minds Centre, Department of Culture and Society, Aarhus UniversityAarhus, Denmark; ^2^Center for Semiotics, Department of Aesthetics and Communication, Aarhus UniversityAarhus, Denmark; ^3^CFIN, Aarhus University HospitalAarhus, Denmark

**Keywords:** heart-beat complexity, exercise, BPM, R-R interval, detrended fluctuation analysis, recurrence quantification analysis

## Abstract

Lately, growing attention in the health sciences has been paid to the dynamics of heart rate as indicator of impending failures and for prognoses. Likewise, in social and cognitive sciences, heart rate is increasingly employed as a measure of arousal, emotional engagement and as a marker of interpersonal coordination. However, there is no consensus about which measurements and analytical tools are most appropriate in mapping the temporal dynamics of heart rate and quite different metrics are reported in the literature. As complexity metrics of heart rate variability depend critically on variability of the data, different choices regarding the kind of measures can have a substantial impact on the results. In this article we compare linear and non-linear statistics on two prominent types of heart beat data, beat-to-beat intervals (R-R interval) and beats-per-min (BPM). As a proof-of-concept, we employ a simple rest-exercise-rest task and show that non-linear statistics—fractal (DFA) and recurrence (RQA) analyses—reveal information about heart beat activity above and beyond the simple level of heart rate. Non-linear statistics unveil sustained post-exercise effects on heart rate dynamics, but their power to do so critically depends on the type data that is employed: While R-R intervals are very susceptible to non-linear analyses, the success of non-linear methods for BPM data critically depends on their construction. Generally, “oversampled” BPM time-series can be recommended as they retain most of the information about non-linear aspects of heart beat dynamics.

## Introduction

As many other physiological processes, heart beat activity has long been considered a process that strives for equilibrium, making regularity indicative of a healthy heart (West, 2006). However, just like many other behavioral and physiological processes, heart beat activity is in fact highly irregular, even during rest (Van Orden et al., 2011). Recent lines of research in physiology even suggest that irregularity in heart beat activity is crucial for health and fitness, turning conventional wisdom on its head. For example, using *Detrended Fluctuation Analysis* (DFA), Peng et al. (1995) showed that heart beat activity in healthy participants follows a fractal pattern called 1/f^α^ noise or *pink noise*. Fractal geometry (Mandelbrot, 1997) seeks to quantify the “roughness” of a surface, but the concept is equally applicable to time-series data: If a process shows strong fractal characteristics, it does not adhere to equilibrium around any specific scale (e.g., a heart rate of 90 beats per min).

Expanding on the findings of Peng et al. (1995), Goldberger et al. (2002) show that heart beat activity of patients with congestive heart failure and atrial fibrillation deviated from healthy “pink” heart rate, in that heart beat fluctuations were less complex, either being less “rough” in the fractal sense or being indeed characterized by an equilibrium, albeit an unhealthy one.

Another complexity metric (or set of metrics) that has gained increasing popularity for the analysis of physiological processes and heart beat activity is Recurrence Quantification Analysis (Marwan, 2013a,b). This time-series analysis technique originates from a chaos-theoretical perspective and is based on phase-space reconstruction of time-series: Takens (1981) showed that the full dynamics of a system of coupled variables can be reconstructed from a single, one-dimensional time-series of that system's behavior.

Recurrence Quantification Analysis has, for example, been used to predict the onset of epileptic seizures: Based on the electro-cardiogram (ECG) record of a patient, a clear pre-seizure change in the ECG was shown at about half a minute prior to seizure onset (Zbilut et al., 2002). Similarly, Wessel et al. (2003) showed that recurrence analysis performed on R-R interval data from a patient who suffered from cardiac arrhythmia predicted the onset of ventricular tachycardia. Beyond potential clinical applications, RQA of heart beat activity has gained increasing popularity in the social sciences, for example as an index of group membership during significant social interactions (Konvalinka et al., 2011).

Even though they quantify different aspects of heart beat dynamics, both techniques (recurrence and fractal analyses) share conceptual similarities in that they are most powerfully applied to measurements that stem from interaction-dominant (Bak, 1996) or coupled component systems (Takens, 1981). Hence, these methods excel at quantifying phenomena that are characterized by the simultaneous presence of structure and irregularities, a mixture that is a natural behavioral outcome of systems that are characterized by interdependencies. Furthermore, both techniques capitalize on variability: Fractal analysis seeks lawful relationships between magnitude and frequency of variability over different (time-) scales, and Recurrence Quantification Analysis uncovers different kinds of dynamic structures. In other words, these non-linear analysis techniques rely on variability that is often viewed as unwanted noise from the perspective of linear statistics—for example when a reliable estimate of average heart rate is pursued. As a consequence, when this noise is removed through data processing or data collection procedures in order to improve the estimation of central tendencies, the result might be a considerable loss of sensitivity of non-linear statistics.

Accordingly, the aim of this study is twofold: First, to investigate whether non-linear statistics (Fractal and Recurrence Analysis) show greater sensitivity for assessment of heart beat activity when compared to the simple “level” of heart rate. Second, to compare the extent to which two standard heart beat measurements, the *beat-to-beat interval* (R-R interval) and the *beats-per-minute* (BPM), differ in the degree to which they contain information about the heart beat dynamics. It is generally to be expected that in order to utilize the full statistical power of non-linear methods—which capitalize on the variability of a signal—the R-R interval should be preferred over standard BPM data. However, since the potential problem with BPM data lies in the smoothing implicit in its construction, we also test two alternative versions of BPM construction in order to assess to what extent heart beat variability can be retained in a BPM signal and whether this has a significant positive impact on the non-linear statistics.

## Experiment and hypothesis

BPM is assumed as the standard statistic for clinical practice (Moody, 2013) and accordingly it is the only output of several devices for the collection of heart beat activity. Furthermore, some studies might critically depend on the retained temporal structure of heart rate time-series characteristic of BPM (but not R-R interval) for the correlation with other time series data (see e.g., Konvalinka et al., 2011). However, the BPM measure is in fact a smoothed heart beat profile due to the moving window averaging that is inherent in its construction. R-R intervals, on the other hand, preserve the natural variability of heart rate activity that is lost in BPM.

In order to assess the impact of the type of underlying time-series data (R-R interval vs. BPM) on the assessment of linear (average HR) and non-linear (Fractal and Recurrence structure) characteristics of heart beat activity, we set up a simple exercise experiment. Participants were asked to sit and rest, then cycle (light exercise), and rest again after exercise. Each phase (rest, exercise, and rest) took 15 min. A review of the literature regarding the impact of exercise or other sources of physiological arousal on Fractal and Recurrence characteristics of heart beat activity yielded the following predictions, summarized in Table 1, together with the expected changes in average heart rate.

**Table 1:** **Expected changes in heart beat activity patterns**.

**Measure**	**Pre-rest-phase**	**Cycling-phase**	**Post-rest-phase**	**References**
Number of beats per minute	Low	High	Low	e.g., Burton et al., 2004
Scaling exponent	Low	High	Low	Baumert et al., 2006; Aoyagi et al., 2007; Busha, 2010; Castiglioni et al., 2011
Recurrence measures of dynamics stability and complexity	Low	High	Low	Mohr et al., 2002; Liu et al., 2004

Summary of expected changes in heart rate level and dynamics during rest and exercise, as reported in other research articles on related topics.

While the average heart beat interval should decrease during cycling (corresponding to an increased BPM), the long-range correlations of heart beat activity (as measured by the fractal scaling exponent) should increase during cycling. Similarly, the general dynamic stability and complexity of heart beat activity (quantified by the RQA measures of %Recurrence, %Determinism, MeanLine, MaxLine and L-Entropy) are expected to increase during cycling compared to rest. %Recurrence captures the overall repetitiveness of the signal, %Determinism captures the extent to which the signal repeats itself in adjacent trajectories, MeanLine captures the average length of these trajectories and MaxLine captures the maximum length of these trajectories. Finally, L-Entropy is the information entropy of the distribution of repeating trajectories and captures the complexity of heart beat activity^1^.

Crucially, we expect higher sensitivity of non-linear measures applied to R-R interval data compared to standard BPM data. Furthermore, we explore the possibility to recapture some of the non-linear properties of alternative, “oversampled” constructions of the BPM data (see the Data Analysis section for details).

## Participants

Seven participants (all male students from the University of Aarhus ranging from 22 to 34 years of age) were included in the study. In accordance with our inclusion criteria, all participants were healthy volunteers with no history of any respiratory or cardiovascular disease and were not taking any medication at the time of the study. Furthermore, all participants were required to have a minimum level of fitness, and exercise regularly (1–6 h a week): Participants in the sample reported to exercise on a weekly basis between 3 and 6 h. Since we recruited a relatively homogeneous sample of participant and all analyses rested on within-subject comparisons, the sample size was deemed to be sufficient given the expected effect sizes reported in the literature (see Table 1). Furthermore, we conducted both sensitivity and *post-hoc* power analyses to assess the appropriateness of the statistics.

To minimize external influences on the autonomic nervous system, participants were asked to consume a light meal no later than 2 h (main meals and dairy products 4 h) prior to the study. They were likewise asked to refrain from smoking, alcohol, and caffeine in the 12 h prior to testing, and no physical exercise was permitted 24 h before testing. Furthermore, participants were not allowed to talk during the experimental sessions.

## Apparatus

A conventional commercial home-cycle was used for exercise task at a low level of intensity. Polar Team2 (Polar, 2013) chest-strapped heart rate monitors were used to record participants' heart beat activity as R-R intervals with millisecond accuracy (as specified by the manufacturer)^2^.

## Procedure

Upon arrival, the measurement device (the Polar Team2 heart rate monitor) was attached to participant's chest and baseline blood pressure (Normal ECG) was taken. Then, participants received instructions about the experiment and filled in forms and questionnaires (consisting of demographic questions, questions regarding health status, as well as written consent). This gave participants' heart beat activity time to settle down. After that, the experimental protocol started and participants were asked to rest in a sitting position on a chair for 15 min. Then, participants were asked to step up on the home trainer right next to their chair and cycle lightly for 15 min. Subsequently, participants were asked to step down from the cycle and rest again in the chair for 15 min. Finally, participants were debriefed.

## Data analysis

**Figure 1:**
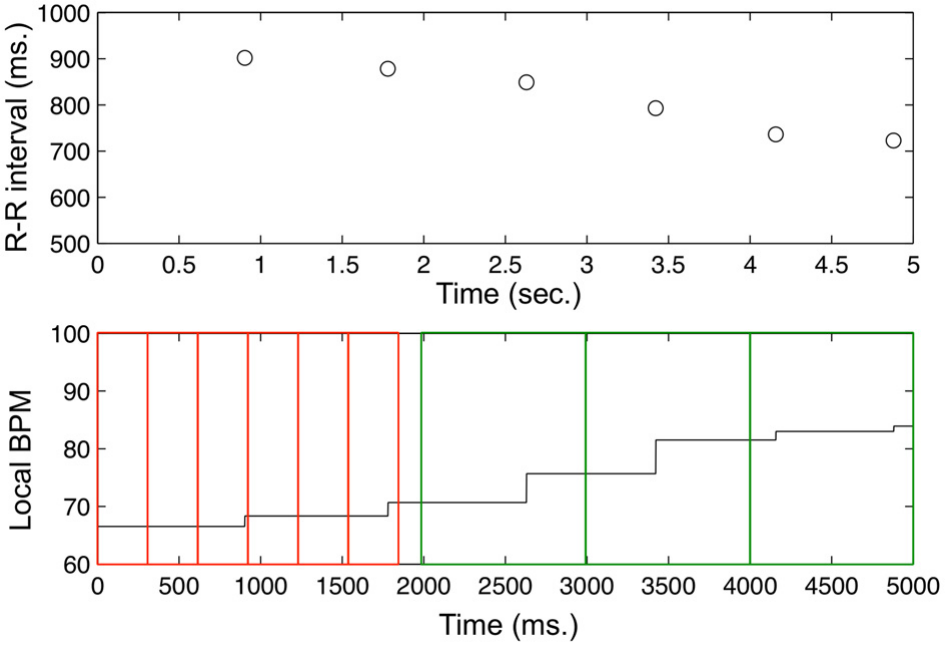
**The top row displays one participants R-R intervals during the first 5 s of rest**. The bottom row display the reciprocal of the R-R intervals sampled at milliseconds and scaled to BPM of the same record. For example, in order to construct BPM data at the level of 0.3 s, this data series is split up in non-overlapping windows of 300 ms over which the level of beats-per-minutes is averaged (displayed by the slim red windows on the left hand side of the lower panel). To construct BPM data at the level of 1.0 s intervals, this window is enlarged to 1000 ms (exemplified by the big green windows on the right hand side of the lower panel). To construct BPM data at the level of 6.0 s intervals, this window would need to be further enlarged to 6000 ms and would be shifted across the data series in 1000 ms steps (not displayed).

Analyses were performed on the R-R interval data obtained from the Polar heart beat monitor and on corresponding BPM data series which were constructed from the R-R intervals. Three different BPM measures were calculated: the reciprocal of each of the R-R interval series was averaged over non-overlapping intervals of 6, 1 and 0.3 s, respectively. Figure 1 illustrates the process.

**Figure 2:**
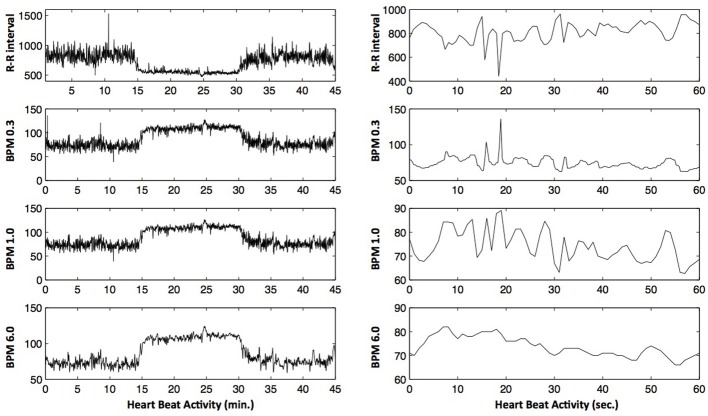
**Example heart beat data of one participant throughout a whole rest-exercise-rest session. Left panel**: Example of one participant's RR-interval-series (top) and the corresponding BPM-data (BPM0.3, BPM1.0, and BPM6.0). **Right-panel**: A close-up of the heart beat data for the first minute of the pre-resting phase. As can be seen, the BPM0.3 data retains a lot of the variability seen in R-R intervals, while most of this variability is lost in the BPM6.0 data.

Following the output option for BPM of the Polar Team2 software we discarded the decimals in the moving window of 6-s, while they were kept in the moving windows of 1 and 0.3 s. Employing three measures allows us to test the impact of the smoothing on the variability of the data: The smaller the window size for the calculation of the BPM series, the weaker the smoothing. Similarly, the information retained in the decimals will equally diminish the influence of the smoothing process and keep more of the variability of heart beat activity that is crucial for non-linear analysis techniques (Figure 2 illustrates three different BPM series together with the resulting data). Roughly speaking, the three BPM series correspond to three somewhat different kinds of data: The BPM series that results from a moving 6-second-window (BPM6.0) is a highly smoothed record of heart beat activity, while the BPM series resulting from the non-overlapping 1-s window (BPM1.0) is substantially less smoothed, and corresponds more to a down-sampled version of the RR-intervals. Finally, the BPM series of non-overlapping 0.3-s intervals (BPM0.3) is similar to an interpolation between RR-intervals, oversampling—instead of smoothing out—heart beat variability.

For each participant's record we separated each phase (pre-rest, cycling, post-rest) by removing the first and last 100 data points (equaling at about 1 min of recording) of each phase before and after transition into the next phase. This was done in order to minimize the impact of the transient changes from one phase into the next.

For the estimation of fractal scaling exponents, expressed as *Hurst exponents*, we used a custom MatLab script to conduct *Detrended Fluctuation Analysis* (DFA), which is the current standard for estimating scaling properties of heart beat activity (Bravi et al., 2011). The analysis was conducted with the following parameter settings: Removal of linear trends in the DFA-detrending-process, minimum bin size of 4 and maximum bin size equal to ¼ of the maximum length of the data set. We followed the recommendations of Holden (2005) for data preparation. Before subjecting the data to analysis, we determined the profile of the data—fractional Gaussian noise (fGn) vs. fractional Brownian motion (fBm)—to select the correct method for the calculation of the Hurst exponents: Data with a fGn profile needs to be integrated before subjection to DFA, while this is not the case for fBm-type data. We examined the power spectral density (PSD) of each data series and determined their profile by plotting the powers against their associated frequencies on log-log axes (Delignières and Marmelat, 2013). A regression line is fitted to the scaling region on this plot and the slope of the line estimates the profiles of the data. Slopes between 0 and −1 indicate fGn-type data, while slopes between −1 and −2 indicate fBm-type data. The steepest slope obtained from all data sets was −0.96. Hence, all data was treated as fGn and integrated before subjection to DFA.

For the estimation of recurrence variables we used Norbert Marwan's *Commandline Recurrence Plots software* (Marwan, 2013a,b). The delay and embedding parameters were estimated for each data set by the first local minima of the average-mutual-information function and the false-nearest neighbor function, respectively (Abarbanel, 1996; Webber and Zbilut, 2005). The average parameter values were used across all participants: Delay of 10 and Dimension of 3 for R-R-intervals, Delay of 20 and Dimension of 3 for 6-s and 1-s BPM data, and Delay of 20 and Dimension of 4 for the 0.3-s BPM data^3^. The Euclidean norm for phase-space re-scaling was used in all cases. The radius parameter was set to yield 2% of recurrent points for the first resting phase (pre-rest) for each participant. The same radius was then kept for the analysis of each participants cycling and post-rest phase. When a resulting recurrence plot yielded less than 1% of recurrent points, then the radius parameter was adjusted for that data set to yield 1% of recurrence (Zbilut et al., 2002). This was the case for one time-series set of BPM0.3 data during cycling.

In order to compare the different linear and non-linear statistics across the rest and exercise conditions, we utilized the following analysis strategy: After the averages, scaling exponents, and recurrence measures were computed for each participants during each of the three phases (pre-rest, cycling, post-rest), the moments were subjected to a repeated-measures ANOVA (SPSS 13, 2013) with the within-subject factor “exercise” (3 levels: pre-rest, exercise, post-rest) for each of the four data series (RR, BPM6.0, BPM1.0, BPM0.3) separately. Follow-up comparisons were made using paired-sample *t*-tests in order to assess the exact location of the global effects (only *p*-values are presented). *Post-hoc* power analyses were conducted to assess the appropriateness of the statistics (Faul et al., 2007). Finally, we used the measure of partial Eta^2^, which reports the proportion of variance that is uniquely explained by the independent variable to investigate the change of statistical power across the measures for the different data types (R-R intervals, BPM6.0, BPM1.0, and BPM0.3).

## Results

### Sensitivity analyses

Pearson correlation coefficients between repeated measures were above 0.180 in all cases except for L-Entropy in BPM1.0 and BPM6.0, for which they were *r* = 0.007 and *r* = 0.011, respectively. A sensitivity analysis for a sample of 7 participants revealed that effects above *F* = 4.45 will be reliably detected. *Post-hoc* power analyses are therefore only reported for effects smaller than this.

### Average heart rate

**Figure 3:**
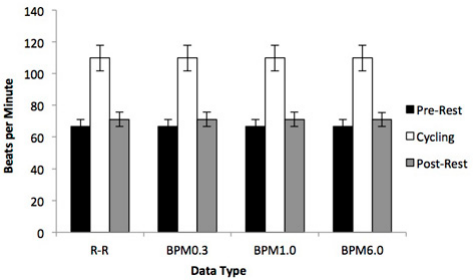
**Average heart rate for R-R intervals, BPM6.0, BPM1.0, and BPM0.3**. As can be seen, overall heart rate increases during exercise. Note that R-R intervals are presented in beats per minute.

Figure 3 presents the average length of RR-intervals and the average number of BPM for all three BPM-series. As expected, all four data series yield a clear increase in heart rate for the cycling phase, expressed as higher BPM and lower R-R, compared to the pre-resting and post-resting conditions [all *F*_(2, 12)_ > 26.93, all *p* < 0.001].

*Post-hoc* tests confirmed that the effect of exercise was due to differences between the two resting phases and the cycling phase (all *p* > 0.001), while the pre- and post-resting phases yielded similar levels of heart rate (all *p* > 0.292).

### Detrended fluctuation analysis

**Figure 4:**
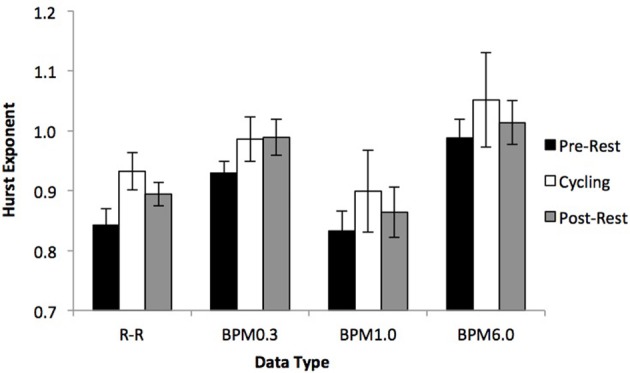
**Average Hurst exponents for R-R intervals, BPM6.0, BPM1.0, and BPM0.3**. For R-R interval and BPM0.3 data, the Hurst exponents increase from pre-rest to exercise and stay on an elevated level during post-rest. No effects are apparent in the BPM1.0 and BPM6.0 data.

Figure 4 presents the average Hurst exponents of R-R intervals and the three BPM-series. While RR-intervals [*F*_(2, 12)_ = 6.51, *p* = 0.006] and BPM0.3 [*F*_(2, 12)_ = 4.06, *p* = 0.045] reveal a reliable effect of exercise on the fractal structure of heart beat activity, no such effect is apparent in the BPM6.0 and BPM1.0 data [both *F*_(2, 12)_ < 0.78, both *p* > 0.418]. *Post-hoc* power analyses reveal a 100% chance to detect this effect in BPM0.3 and a 75.1% chance to detect it in BPM6.0 and BPM1.0.

*Post-hoc* tests for the RR-interval and BPM0.3 data revealed that the difference in Hurst exponents was located in the transition from pre-rest to cycling (both *p* < 0.012), while the observed Hurst exponents during post-rest were equally high compared to those observed during cycling (both *p* > 0.272). Hurst exponents of heart beat activity during post-rest were also reliably higher compared to pre-rest in RR-intervals (*p* = 0.012) and still marginally higher compared to pre-rest in BPM0.3 (*p* = 0.066).

### Recurrence quantification analysis

**Figure 5:**
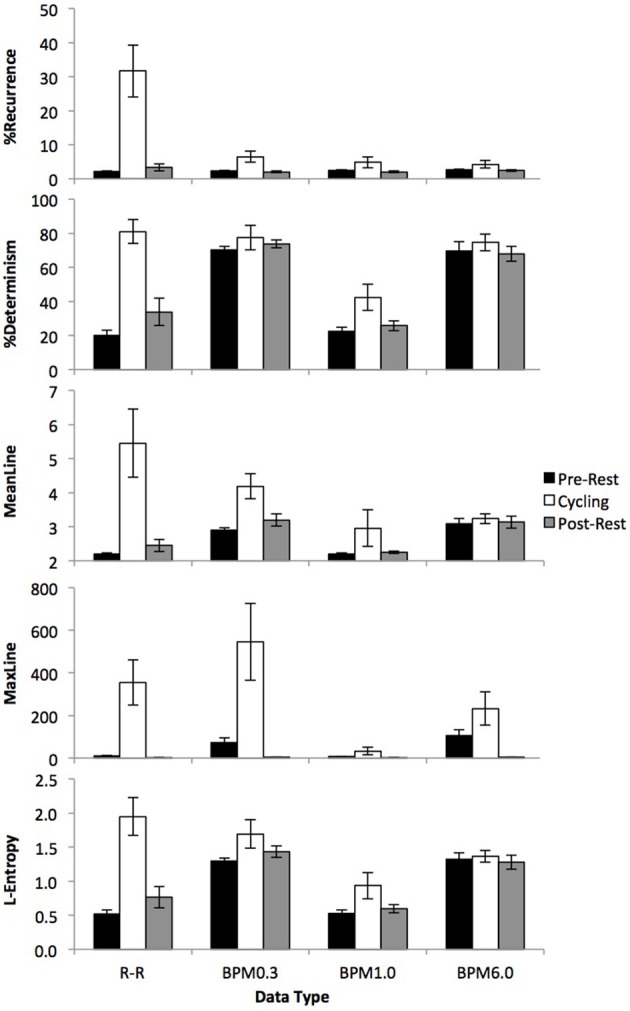
**Average %Recurrence (top), %Determinism (2nd to top), MeanLine (3rd to top), MaxLine (4th to top) and L-Entropy (bottom) for R-R intervals, BPM0.3, BPM1.0, and BPM6.0**. For R-R intervals, all recurrence measures generally increased during exercise compared to rest. The statistical differences between rest and exercise are somewhat diminished for BPM0.3, and are generally absent in the BPM1.0 and BPM6.0 data (with the exception of %Determinism for the BPM1.0 data).

Figure 5 presents the differences in recurrence measures between rest and cycling for RR-intervals, and the BPM6.0, BPM1.0, and BPM0.3 data.

For RR-intervals, we observed a consistent increase in all five recurrence measures for the cycling phase compared to the rest phases [all *F*_(2, 12)_ > 9.05, all *p* < 0.002].

For the BPM0.3 data, we observed an increase of %Recurrence, MeanLine and Maxline during cycling [all *F*_(2, 12)_ > 5.95, all *p* < 0.016), but no differences in %Determinism or L-Entropy [both *F*_(2, 12)_ < 1.36, both *p* > 0.298). *Post-hoc* power analyses reveal a 97.7% chance to detect such effects; thus, it is very unlikely that our negative findings in this case can be attributed to sample size.

For the BPM1.0 data, we observed an increase of %Determinism during cycling [*F*_(2, 12)_ > 4.98, *p* = 0.027], but no effect on any of the other measures [all *F*_(2, 12)_ < 2.64, all *p* > 0.112]. Also, there were no effects of exercise on any of the recurrence measures for the BPM6.0 data [all *F*_(2, 12)_ < 2.67, all *p* > 0.110]. *Post-hoc* power analyses reveal a 100% chance to detect such effects in both BPM1.0 and BPM6.0. Again, it is very unlikely that the negative findings can be attributed to low sample size.

If an effect of exercise on any of the recurrence measures was observed, then *post-hoc* tests confirmed that the effect was due to differences between the two resting phases and the cycling phase (all *p* > 0.051), while the pre- and post-resting phases yielded similar levels of heart dynamics (all *p* > 0.159).

### Statistical model comparison

**Figure 6:**
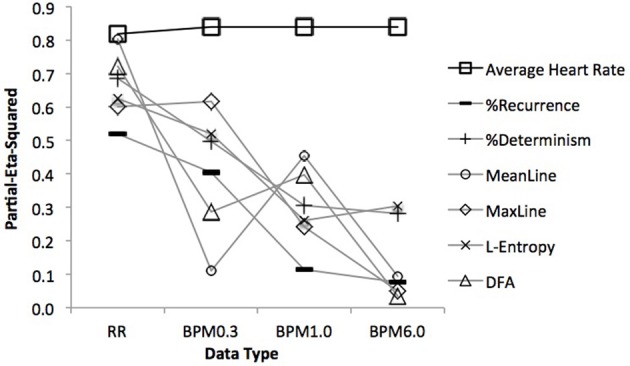
**Relationship between explained variance and data type for average heart rate and the complexity metrics**. As can be seen, complexity metrics work best for R-R intervals and generally less well for BPM-type data. The power of the non-linear statistics decreases with growing window sizes over which the BPM data is constructed. This is not the case for average heart rate.

Figure 6 depicts the explained variance (partial Eta^2^) of the statistical models as a function of data type (R-R, BPM0.3, BPM1.0, BPM6.0). As can be seen, the overall level of heart rate (black line) remains virtually unaffected by the choice of data type, while the complexity measures (gray lines) loose statistical power as the moving window size for the construction of the BPM-series is increased.

## Discussion

The results of our study show that active exercise leads to an increase in heart rate which is necessary to increase the supply of oxygen and nutrients for the muscles and other participating organ systems (e.g., Burton et al., 2004). While this result might seem trivial, we were also able to produce a more articulate picture of the effects of exercise with the help of complexity metrics. Interestingly, these effects could not be observed in standard preparations of BPM data, but only when the analysis was based on R-R intervals or “oversampled” BPM data (BPM0.3). Accordingly, we observed a clear loss of statistical power of the complexity metrics in the BPM-data as a function of the window size over which the BPM data was constructed.

In addition to expected increase of heart rate during exercise compared to the two rest periods, we observed a general increase in the dynamic stability of heart beat activity during exercise, as evidenced by an across-the-board increase in recurrence measures of %Recurrence, %Determinism, MeanLine and Maxline. These observed changes in the recurrence measures of heart beat activity might capture the increase of constraints on heart beat variability during exercise compared to rest, which can be interpreted as the exercise condition creating a relatively stronger attractor for heart beat fluctuations (e.g., Richardson et al., 2007).

The final two measures, L-Entropy and the Hurst exponent add to these findings indicating that heart beat fluctuations became also more complex during exercise. This might indicate a stronger coupling of heart beat activity to other relevant physiological processes, such as breathing (Hirsch and Bishop, 1981). As the interdependencies to other physiological processes increase, heart beat activity becomes more complex since the activity of other physiological systems is now more strongly reflected in the heart beat dynamics in addition to the intrinsic dynamics of the heart itself.

Particularly the gravitation of the fractal scaling exponent toward *H* = 1 indicates a kind of complexity that could stem from an increase in interaction-dominant dynamics that result form the (successful) coordination of multiple physiological components or processes (Bak, 1996; Goldberger et al., 2002; Webber, 2005). Interestingly, while the classical measure of heart rate level as well as the non-linear recurrence measures classify the two resting phases as similar (but different from the exercise phase), the fractal scaling properties of heart beat activity are different from the pre-resting phase to exercise, but remain similar to exercise during post-rest. This could be an example of known long-term carry-over effects in heart rate variability (e.g., Akselrod et al., 1981; Terkelsen et al., 2012): A kind of hysteresis effect that governs long-term development of physiological processes (such as the long-term changes of heart physiology and activity patters through repeated-short-term events, as for instance exercise) and is better detectable by complexity metrics compared to linear measures, such as the overall level of activity or linear power-spectral density analysis (Webber et al., 2009). The result is supported by findings of Karavirta et al. (2009) indicating that long-term exercise gains in older participants of endurance and strength training went together with increased fractal scaling exponents of heart beat activity. However, while this effect was shown on a time-scale of several weeks, we observe comparable effects on a much shorter time-scale.

Importantly, these results could not unanimously be obtained from the BPM data series: While all four kinds of heart beat activity time series (RR, BPM6.0, BPM1.0, BPM0.3) showed a clear effect of the overall level of heart rate, the two BPM-series with down sampled and (heavily) smoothed data yielded no results on the complexity metrics obtained from recurrence and fractal analysis (the exception being a comparatively small effect of exercise on %Determinism in the BPM1.0 data).

In sum, while overall level of heart rate distinguishes well between the exercise and rest periods, it is blind to the more subtle relations between exercise, pre- and post-rest periods. Non-linear statistics relying on R-R intervals appear to have the highest sensitivity in describing heart rate dynamics, followed by non-linear statistics on BPM decreasing in proportion to down-sampling/smoothing. However, when the BPM data was constructed in such a way as to over-sample the RR-intervals (the BPM0.3 data), most of the effects observed in the RR-intervals could be observed in the BPM data as well, even though the overall strengths of the effects was somewhat weaker. Thus, the oversampled BPM0.3 measure seems to be a better solution for studies that depend on evenly spaced measurements, for example to correlate heart beat with other time series data (e.g., Konvalinka et al., 2011).

In order to adequately assess heart beat dynamics, it is highly recommendable to use non-linear statistics on R-R intervals, which boosts the statistical power of non-linear statistics. Crucially, non-linear statistics—such as Recurrence Quantification Analysis and DFA—grant a more detailed perspective on temporal dynamics.

This study was intended as a proof-of-concept aimed at comparing the sensitivity of different methods and measures of heart rate reported in the literature. Our results suggest a considerable detrimental effect of smoothing associated with BPM for the estimation of non-linear aspects of heart rate variability. At the same time, it points to the high potential of non-linear statistics applied to R-R intervals or over-sampled BPM. Even considering the small number of participants, the statistical power of the analyses shows that R-R intervals are a data type that yields very reliable effects, and it is unlikely that the lack of effects in the analyses involving BPM1.0 and BPM6.0 data is due to the limited sample size. Taken together, this motivates important methodological considerations for the recording and analysis of heart rate data for research that aims at systematic investigations of the role of heart rate fluctuations in exercise, fitness, aging, and wellbeing.

### Conflict of interest statement

The authors declare that the research was conducted in the absence of any commercial or financial relationships that could be construed as a potential conflict of interest.

## Footnotes

^1^Recurrence Quantification Analysis offers many outcome variables that could quantify changes in heart beat in this experiment. However, we chose only to present the statistics, %Recurrence, %Determinism MeanLine, MaxLine and L-Entropy statistics. This was done for the following reasons: First, these five statistics are the ones most commonly used in the literature (Giuliani et al., 1998; Marwan et al., 2002; Wessel et al., 2003; Acharya et al., 2011). Second, the effects observed in these five measures are redundant with the effects observed in other recurrence variables (i.e., LAM, TT, VMAX, VENTR).

^2^The mobile devices for the measurement of R-R intervals by Polar have generally show excellent agreement with measurements obtained from ECGs (e.g., Laukkanen and Virtanen, 1998; Vanderlei et al., 2008; Weippert et al., 2010).

^3^We tested the effects over a variety of parameter setting, using delays between 1 and 20 and dimensions between 3 and 6 for R-R intervals, as well as delays between 10 and 30 and dimensions between 3 and 6 for the three BPM-series. The overall results remained stable across the different parameters.
